# Hepatic iron accumulation is reduced in the cholestatic *Mdr2^−/−^* mouse

**DOI:** 10.1042/BSR20250360

**Published:** 2026-07-22

**Authors:** Amy L. Sobbe, Kim Bridle, Lesley-Anne Jaskowski, David M. Frazer, Gautam Rishi, Afolabi Akanbi, Gregory Anderson, Darrell H.G. Crawford, V. Nathan Subramaniam

**Affiliations:** 1 Faculty of Health, Medicine and Behavioural Sciences, The University of Queensland, Brisbane, Australia; 2 Gallipoli Medical Research, Greenslopes Private Hospital, Brisbane, Australia; 3 The QIMR Berghofer Medical Research Institute, Brisbane, Australia; 4 Hepatogenomics Research Group, Centre for Genomics and Personalised Health, School of Biomedical Sciences, Queensland University of Technology (QUT), Brisbane, Queensland, Australia

**Keywords:** cholestasis, hepcidin, iron dysregulation, Iron metabolism, liver, MDR3

## Abstract

The multidrug-resistance 2 (*Mdr2^−/−^*) mouse is an animal model of biliary liver injury and fibrosis. This genetic equivalent of the human disorder progressive familial intrahepatic cholestasis type 3 histologically resembles primary sclerosing cholangitis. Bile acid accumulation during cholestasis is linked to the down-regulation of the iron regulatory hormone hepcidin, thus suggesting a link between liver disease and iron homeostasis. In the present study, we investigated iron homeostasis in the *Mdr2^−/−^* mouse model of cholestasis. Iron levels and expression of iron-related genes were analysed by real-time PCR and western blotting. Accumulation of iron and the hepcidin response were analysed in wild-type and *Mdr2^−/−^* mice challenged with either an iron-deficient or a 1% carbonyl iron diet. *Mdr2^−/−^* mice on a control diet had reduced hepatic iron stores when compared with age-matched controls, despite lower hepatic hepcidin expression and a corresponding elevation in hepatic transferrin receptor 1 expression. *Mdr2^−/−^* mice fed a 1% carbonyl iron diet were resistant to hepatic iron accumulation, despite increased serum iron, suggesting impaired hepatocyte iron uptake in this model. In conclusion, *Mdr2^−/−^* have abnormal hepatic iron homeostasis, potentially resulting from cholestasis. Impaired hepatic iron uptake may explain the relative paucity of liver iron in cholestatic compared with hepatocellular conditions.

## Introduction

Alterations in iron homeostasis (particularly hepatic siderosis) are frequently seen in chronic liver disease independent of genetic hemochromatosis, and those with high hepatic iron are likely to display more advanced fibrosis, increasing the risk of hepatocellular carcinoma and leading to more adverse outcomes following orthotopic liver transplantation [1–4]. Patients with alcoholic liver disease (ALD), viral hepatitis, and non-alcoholic steatohepatitis (NASH) often have transferrin saturation (TS) and serum ferritin levels suggestive of haemochromatosis [5–7] even though they lack mutations that predispose to the disorder. Ludwig et al. [8] described positive iron staining in 145 biopsies (32.4%) from patients with cirrhosis (but haemochromatosis was the primary aetiology in only five of these patients). Stuart et al. [9] showed similar results in a study of 282 liver explants (although only seven patients had a pre-transplant diagnosis of hereditary haemochromatosis). Positive iron staining was seen in 35% of non-HH patients with liver disease, and 9.5% of total liver explants had Grade 3 or 4 iron accumulation.

Iron deposition in cirrhosis occurs predominantly in hepatocytes, although proliferating bile ducts often contain stainable iron [9]. RE (Kupffer) cell iron has also been reported, and this is associated with more severe disease in NASH, hepatitis C viral infection, and ALD [10–12]. The cause of hepatic siderosis in advanced liver disease is poorly understood but is likely to be multifactorial. Spur cell haemolytic anaemia is a feature of advanced cirrhosis and results from the incorporation of free cholesterol into the erythrocyte membrane, leading to an increased surface-area-to-volume ratio and spur-like projections [13]. The presence of spur cells has been linked to increased hepatic iron stores [11,14–16]. These cells have a reduced lifespan, thereby increasing the erythropoietic demand, which in turn reduces hepcidin levels and thus increases iron absorption. In keeping with this theory, increased absorption of ^59^Fe has been reported in patients with cirrhosis [17] along with increased expression of the duodenal iron transporters divalent metal-ion transporter 1 and ferroportin 1 (*FPN1*) [18]. Neither of these studies reported haemochromatosis patients being included in their cohort. These duodenal features may also be a result of decreased hepcidin synthesis associated with declining hepatic synthetic function [19]. TF synthesis and, consequently, total iron-binding capacity (TIBC) are also reduced in cirrhosis, leading to an increase in non-transferrin-bound iron (NTBI), which is readily taken up by hepatocytes [8].

Hepatic siderosis is more common in hepatocellular liver injury than it is in cholestatic disease. Ludwig and colleagues [8] demonstrated positive iron staining in 55% of patients with hepatocellular liver diseases (excluding HH), but only in 9% of patients with biliary injury. Thirty-four percent of those with hepatocellular injury had hepatic iron concentrations above the normal range, but this was seen in only 4.5% of patients with biliary cirrhosis. Similar results were described in an Australian study by Stuart *et al.* [9]. Of 156 patients with hepatocellular cirrhosis, 94 (60%) had positive iron staining, whereas only 10 (7.9%) of 126 patients with biliary cirrhosis had stainable hepatic iron. Increased iron absorption and tissue iron deposition are appropriate responses to reduced hepcidin levels. Given the similar degrees of hepatic dysfunction, it is unusual that hepatic siderosis occurs infrequently in cholestatic liver injury when compared with hepatocellular disease. The mechanisms responsible for these differences in iron loading are unclear.

Iron homeostasis has been investigated in the bile duct ligation (BDL) rat model of cholestasis [20]. Rats subjected to BDL had a lower hepatic iron concentration than sham-operated controls as well as reduced expression of the gene encoding hepcidin, *Hamp1*, and approximately three-fold increased plasma iron levels. The low hepatic iron phenotype was attributed to increased hepatic expression of FPN1. Administration of the cholesterol-reducing drug pravastatin abrogated the hepatic iron deficiency; however, plasma iron levels and hepatic FPN1 expression did not return to sham levels. Sanyal et al. [21] have also demonstrated that BDL rats have impaired intestinal iron absorption resulting from the depletion of bile acids following BDL. While these studies have defined altered iron homeostasis in cholestasis, the mechanisms of hepatic iron deficiency have not been completely explored. Likewise, an investigation of how iron homeostasis changes with injury progression would also help to understand any associated pathologic role of hepatic iron deficiency.

Multidrug-resistance 2 (*Mdr2^−/−^*) encodes a canalicular phospholipid transporter and is a mouse ortholog of the human *MDR3* gene. Disruption of the *Mdr2* gene in mice leads to an altered composition of bile acids in the bile, where phosphatidylcholine is completely absent [22,23]. Several studies have reported that the *Mdr2^−/−^* mouse is a reproducible model of spontaneous chronic biliary liver disease associated with fibrosis [24–26].

In the present study, we investigated iron homeostasis in the *Mdr2^−/−^* mouse model of cholestasis at various stages of injury. The mechanisms of hepatic iron deficiency were also studied by feeding wild-type and *Mdr2^−/−^* mice either an iron-deficient or a 1% carbonyl iron diet. Our studies show that *Mdr2^−/−^* mice have reduced hepatic iron stores and that this hepatic iron deficiency may be due to impaired hepatocyte iron uptake.

In addition, the present study describes the first extensive investigation of iron homeostasis in biliary liver injury and has the potential to explain the paucity of hepatic iron loading in patients with cholestasis.

## Results

### Serum iron indices in wild-type and *Mdr2^−/−^* mice

**Figure 1:**
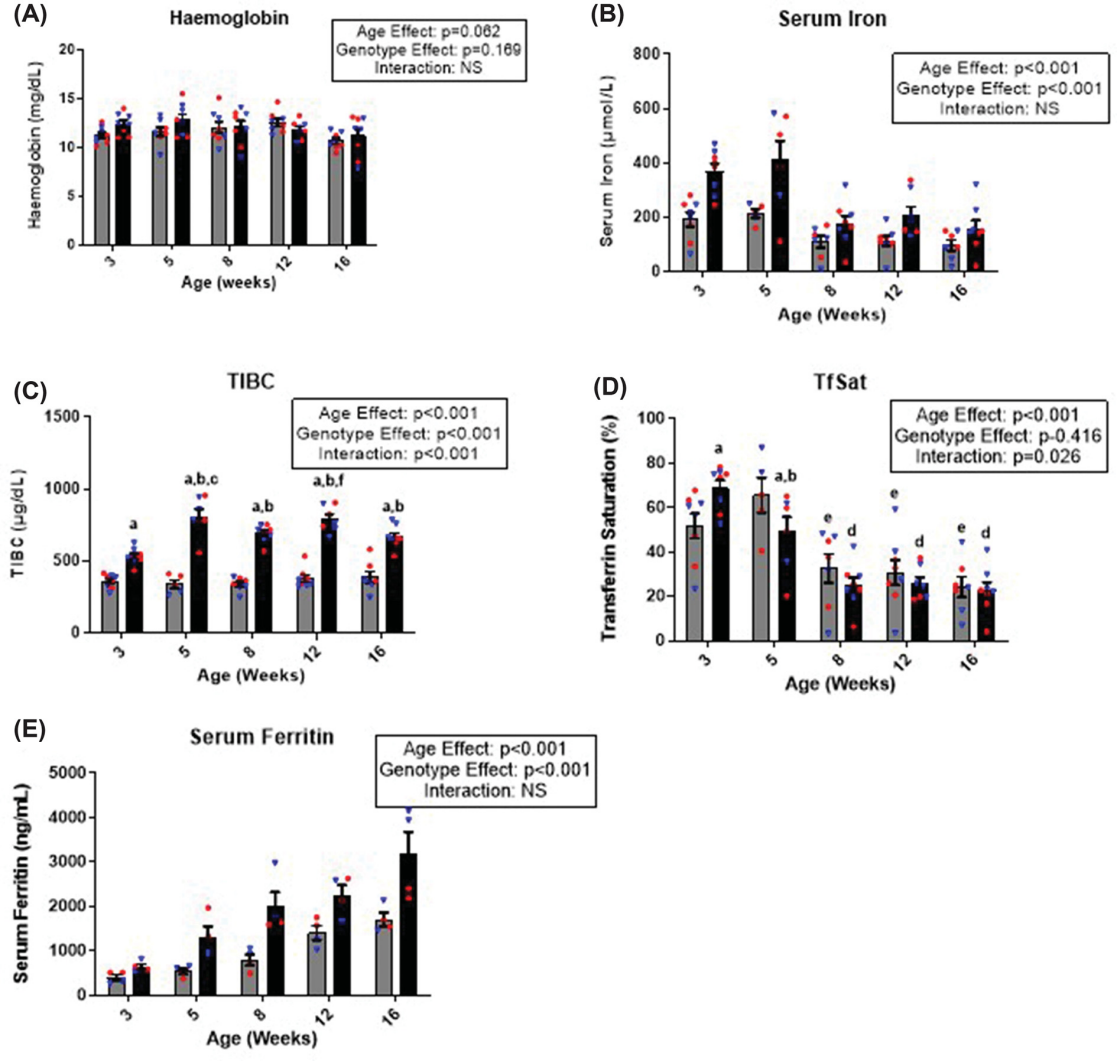
Serum iron indices in wild-type and Mdr2^−/−^ mice (**A**) Haemoglobin, (**B**) serum iron, (**C**) total iron-binding capacity, (**D**) transferrin saturation, and (**E**) Serum ferritin were measured in wild-type and knockout mice at 3, 5, 8, 12, and 16 weeks of age. Results are expressed as mean ± SEM from *n* = 4–8. Least significant differences (LSD) post-hoc analysis was performed when a significant Age*Genotype Interaction was noted: a*P*<0.05 Mdr2^+/+^ versus Mdr2^−/−^ of the same age. b*P*<0.05 versus 3-week-old Mdr2^−/−^. c*P*<0.05 versus 8- and 16-week-old Mdr2^−/−^. d*P*<0.05 versus 5-week-old Mdr2^−/−^. e*P*<0.05 versus 3- and 5-week-old Mdr^+/+^. f*P*<0.05 versus 16-week-old Mdr2^−/−^. Red symbols = male mice; blue symbols = female mice.

To determine whether altered iron homeostasis was a feature of murine models of cholestasis, we first examined serum iron indices in wild-type and *Mdr2^−/−^* mice. Haemoglobin (Hb) levels did not differ significantly between wild-type and *Mdr2^−/−^* mice at any of the ages studied (Figure 1). *Mdr2^−/−^* mice had higher serum iron levels than wild-type controls at all ages; however, there was no significant interaction between age and genotype for serum iron (Figure 1). TIBC was significantly higher in *Mdr2^−/−^* mice than in wild-type mice at all ages (*P*<0.05 at all ages, Figure 1), while TS was significantly different between *Mdr2^−/−^* and wild-type mice at 3 and 5 weeks of age (Figure 1). TS levels declined with age between 5 and 16 weeks of age in both genotypes. Serum ferritin increased with age in both wild-type and knockout mice (Figure 1). In addition, serum ferritin was higher in *Mdr2^−/−^* mice when compared with wild-type controls at all ages, but there was not a significant interaction between age and genotype for serum ferritin (Figure 1). The increases in serum iron, TIBC, and serum ferritin in *Mdr2^−/−^* animals are consistent with cholestasis altering iron homeostasis.

### Hepatic iron stores are lower in *Mdr2^−/−^* mice

**Figure 2:**
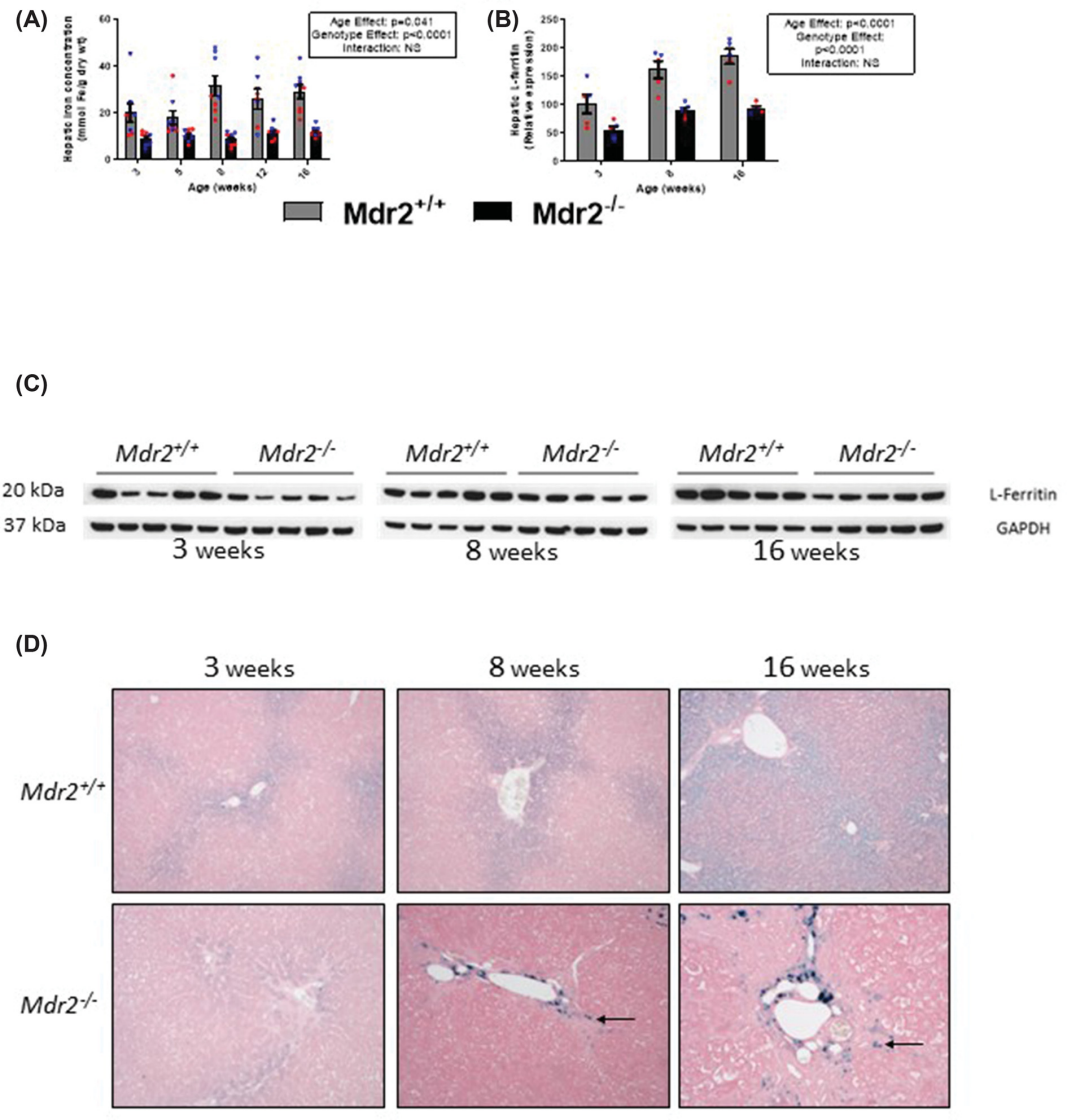
Hepatic iron stores are reduced in Mdr2-/- mice (**A**) Hepatic iron concentration was lower in Mdr2^−/−^ mice from 8 to 16 weeks of age compared with wild-type mice; however there was no statistically significant interaction between age and genotype. (**B,C**) Hepatic L-ferritin levels were assessed by Western blotting and blots were quantified using densitometry. L-ferritin levels increased with age and were lower in Mdr2^−/−^ mice than in wild-type controls at 8 and 16 weeks of age; however, there was no statistically significant interaction between age and genotype. (**D**) Iron distribution in the livers of Mdr2^−/−^ and wild-type mice at various ages. RE macrophages are indicated with black arrows. Representative photomicrographs of tissue samples stained with Perls’ Prussian blue. Original magnification: 100×. Results are presented as mean ± SEM from *n* = 5–8. Red symbols = male mice; blue symbols = female mice. Original magnification: 100×.

Since hepatic iron levels are reduced in human cholestatic liver disease, we next examined hepatic iron stores. The hepatic iron concentration was approximately two- to three-fold lower in *Mdr2^−/−^* mice than in wild-types of the same age, but there was no significant age*genotype interaction (Figure 2A). Likewise, hepatic L-ferritin levels (Figure 2B,C) increased with age and were lower in *Mdr2^−/−^* mice than in age-matched wild-type controls. Perls’ Prussian blue staining of liver sections (Figure 2D) revealed iron deposition within Zone I hepatocytes of wild-type mice from 3–16 weeks of age. In 3-week-old *Mdr2^−/−^* mice, stainable iron was detected in hepatocytes surrounding the portal region; however, staining was less intense than that seen in wild-type animals. At 8 weeks of age, stainable iron was absent from hepatocytes but was detected in reticuloendothelial (RE) cells of *Mdr2^−/−^* mice. Further, RE iron deposition was evident at 16 weeks of age. The mechanism responsible for the pattern of iron deposition in the hepatic RE cells rather than hepatocytes is unclear but may be a result of the necroinflammatory activity present in the *Mdr2^−/−^* model.

Alterations in iron homeostasis in *Mdr2^−/−^* mice were seen from 3 to 16 weeks of age, a time course reflecting the full spectrum of injury including mild periportal fibrosis through to advanced fibrosis and cirrhosis (Supplementary Figure S1). Liver function (alanine aminotransferase, aspartate aminotransferase, and alkaline phosphatase) was significantly up-regulated in knockout animals compared with controls (Supplementary Figure S2). Likewise, hepatic hydroxyproline and expression of fibrogenic genes (procollagen type 1, platelet-derived growth factor receptor B, and tissue inhibitor of metalloproteinase 1) are increased in knockout animals at 3, 5, 8, 12, and 16 weeks (Supplementary Figure S3). This finding suggests that hepatic iron deficiency may be the direct result of Mdr2 absence or cholestasis rather than the liver injury observed in *Mdr2^−/−^* mice.

### Wild-type and *Mdr2^−/−^* mice have similar splenic and cardiac iron stores

To determine whether the pattern of iron loading of other organs was altered in cholestasis, we examined splenic and cardiac iron concentrations and distribution. Splenic iron levels increased with age in wild-type and *Mdr2^−/−^* mice; however, they did not differ with genotype (Supplementary Figure S4A). Cardiac iron concentration was similar in wild-type and knockout animals (Supplementary Figure SB). The age-related increase in splenic iron deposition was confirmed by Perls’ Prussian blue staining (Supplementary Figure S4C), and iron was noted in the red pulp in both wild-type and *Mdr2^−/−^* mice. These results suggest that altered tissue iron loading may be limited to the liver in our model.

### *Mdr2^−/−^* mice have reduced hepcidin expression

**Figure 3:**
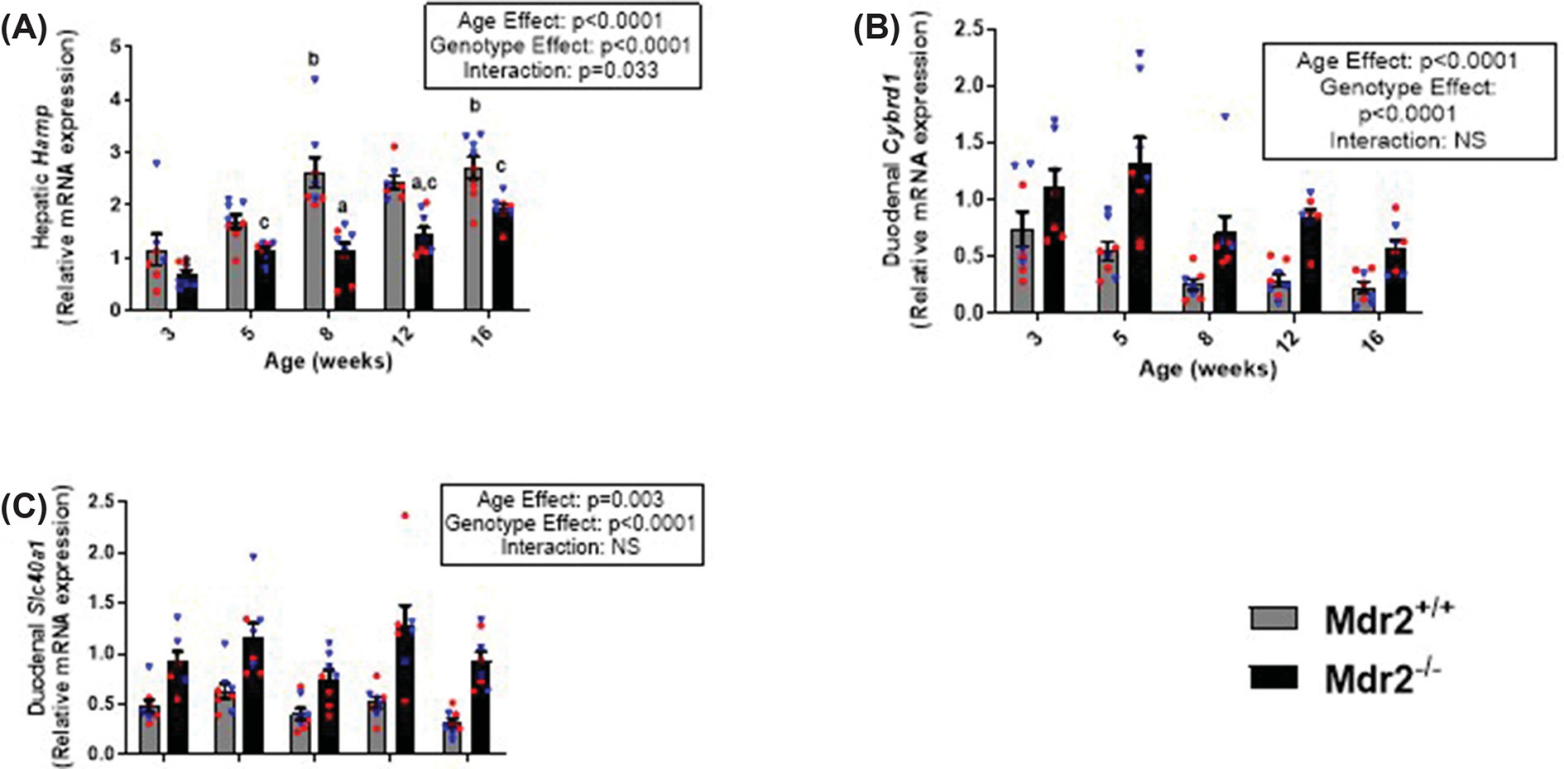
Hepatic Hamp expression is reduced, whereas hepatic Cybrd1 and Slc40a1 expression are increased in Mdr2^−/−^ mice (**A**) Hepatic Hamp expression was significantly lower in Mdr2^−/−^ mice when compared with wild-type mice at 8 and 12 weeks of age, and there was a significant interaction between age and genotype. Cybrd1 (**B**) and Slc40a1 (**C**) were higher in Mdr2^−/−^ mice than wild-type mice of the same age; however, there was no significant interaction between age and genotype for Cybrd1 or Slc40a1. Results are presented as mean ± SEM. *n* = 5–8. a*P*<0.05 versus wild-type of the same age; b*P*<0.05 versus 3-week-old wild-type; c*P*<0.05 versus 3-week-old Mdr2^−/−^. Red symbols = male mice; blue symbols = female mice.

To investigate whether changes in hepcidin expression could contribute to aberrant iron loading in *Mdr2^−/−^* mice, we examined hepatic hepcidin expression and the duodenal expression of *Cybrd1* and *Slc40a1*. Hepatic *Hamp* mRNA expression (Figure 3A) increased in wild-type mice over time. In *Mdr2^−/−^* mice, *Hamp* expression was 1.7-fold, 1.5-fold, 2.3-fold, 1.7-fold, and 1.4-fold lower than that of wild-type mice at 3, 5, 8, 12, and 16 weeks; however, this only reached statistical significance at 8 and 12 weeks. The duodenal expression of both *Cybrd1* (Figure 3B) and *Slc40a1* (Figure 3C) mRNA was higher in *Mdr2^−/−^* mice compared with wild-type mice; however, there was no significant interaction between age and genotype for *Cybrd1* and *Slc40a1*. Taken together, these results are consistent with the reduced hepatic iron observed in the *Mdr2*^−/−^ animals.

### Altered hepatic iron transporter expression in *Mdr2^−/−^* mice

**Figure 4:**
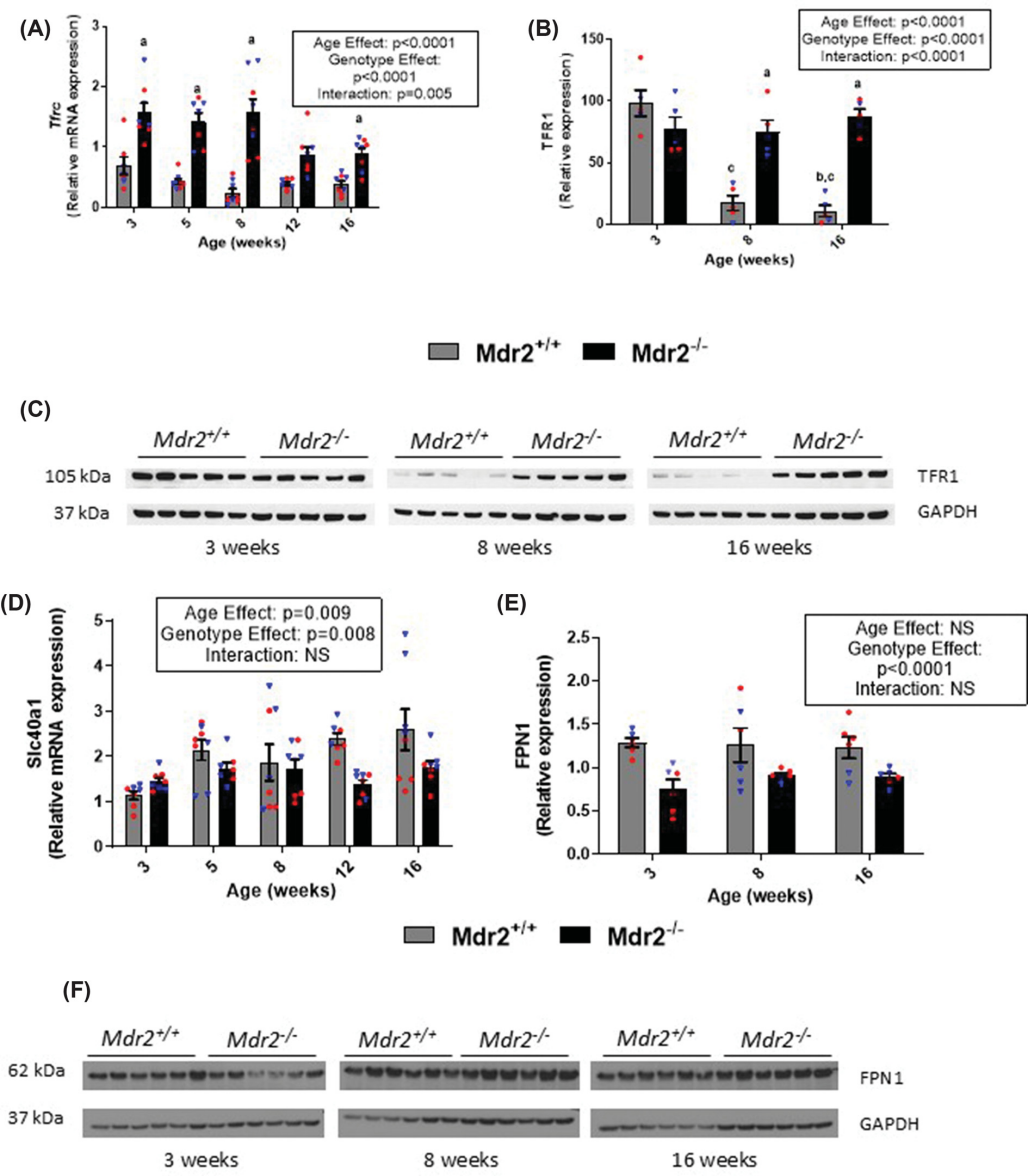
Hepatic iron transporter expression in wild-type and Mdr2^−/−^ mice Hepatic Tfrc mRNA (**A**) and TFR1 protein (**B,C**) expression were higher in Mdr2^−/−^ mice than in wild-type mice of the same age. Slc40a1 mRNA (**D**) and FPN1 protein levels (**E,F**) were not significantly different in wild-type mice. Results are presented as mean ± SEM from *n* = 5–8. a*P*<0.05 versus wild-type of the same age; b*P*<0.05 versus 3 and 8 week old Mdr2^−/−^, c*P*<0.05 versus 3-week-old wild-type. Red symbols = male mice; blue symbols = female mice.

To evaluate if cholestasis induces changes in iron transporter levels, we examined gene and protein expression of transferrin receptor 1 and ferroportin. Hepatic *Tfrc* mRNA expression was significantly higher in *Mdr2^−/−^* mice at 3 (2.3-fold), 5 (3.3-fold), 8 (6.3-fold), and 16 (2.3-fold) weeks of age (Figure 4A) relative to age-matched wild-type controls. TFR1 protein expression (Figure 4B,C) was similar in *Mdr2^−/−^* mice at 3, 8 and 16 weeks of age but was significantly higher than that of wild-type mice at 8 and 16 weeks. Hepatic *Slc40a1* mRNA levels were lower in knockout animals compared with wild-type mice from 5–16 weeks of age (Figure 4D). FPN1 protein levels were lower in *Mdr2^−/−^* mice than in wild-type controls at 3, 8, and 16 weeks of age (Figure 4E,F). The increased transferrin receptor 1 and reduced ferroportin expression in knockout mice were not sufficient to overcome the hepatic iron deficiency in knockout animals.

### Body, liver, and spleen weights and haematological parameters in wild-type and *Mdr2^−/−^* mice fed control, 1% carbonyl iron, or iron-deficient diets

We next sought to challenge the *Mdr2^−/−^* mice with either high-iron or iron-deficient diets to determine whether the response to such iron challenges was similar to wild-type mice. Wild-type and *Mdr2^−/−^* mice were fed control, 1% carbonyl iron, or iron-deficient diets as indicated in the ‘Materials and methods’ section. Body, liver, and spleen weights were recorded for all animals, and the liver and spleen to body weight ratios were calculated (Table 1). Body weight was similar in wild-type mice and *Mdr2^−/−^* mice when fed the same diet. Both liver weight and the liver-to-body weight ratio were significantly higher in *Mdr2^−/−^* mice relative to wild-type mice fed the same diet. *Mdr2^−/−^* mice fed an iron-deficient diet also had a lower liver weight than *Mdr2^−/−^* mice fed a control diet. Both spleen weight and the spleen-to-body weight ratio were higher in *Mdr2^−/−^* mice than in wild-type mice (Table 1).

**Table 1:** Body, liver, and spleen weights in wild-type and *Mdr2^−/−^* mice fed control, 1% carbonyl iron, or iron-deficient diets

Tissue weight	Genotype	Control diet	1% Carbonyl iron	Iron-deficient diet
**Body Weight (g)**	***Mdr2^+/+^***	26.2 ± 0.7	24.9 ± 0.6	25.0 ± 0.6
	***Mdr2^−/−^***	24.3 ± 0.8	23.9 ± 0.6	22.8 ± 0.8
Liver Weight (g)*	***Mdr2^+/+^***	1.36 ± 0.03	1.46 ± 0.05	1.26 ± 0.04
	***Mdr2^−/−^***	2.02 ± 0.11^a^	2.16 ± 0.12^a^	1.57 ± 0.09^ab^
LW:BW (%)*	***Mdr2^+/+^***	5.23 ± 0.11	5.87 ± 0.09	5.02 ± 0.07
	***Mdr2^−/−^***	8.37 ± 0.39^a^	8.99 ± 0.31^a^	6.92 ± 0.32^ab^
**Spleen Weight (mg)**	***Mdr2^+/+^***	103 ± 3.4	102 ± 4.8	228 ± 39.3
	***Mdr2^−/−^***	176 ± 10.4	152 ± 9.2	267 ± 42.2
**SW:BW (%)**	***Mdr2^+/+^***	0.40 ± 0.02	0.42 ± 0.02	0.91 ± 0.16
	***Mdr2^−/−^***	0.74 ± 0.05	0.64 ± 0.03	1.17 ± 0.18

Results are expressed as mean ± SEM from *n* = 4–8. * Significant diet*Genotype interaction *P*<0.05. Post-hoc analysis: ^a^*P*<0.05 *Mdr2^+/+^* versus *Mdr2^−/−^* fed the same diet. ^b^*P*<0.05 compared with *Mdr2^−/−^* mice fed a control diet. LW, liver weight; BW, body weight; SW, spleen weight.

As expected, the Hb of animals on an iron-deficient diet was lower than that of animals on control and carbonyl iron diets; however, there were no significant differences associated with genotype. Serum iron levels were similar in wild-type and *Mdr2^−/−^* mice fed either a control diet or an iron-deficient diet but were significantly higher in *Mdr2^−/−^* mice fed 1% carbonyl iron relative to wild-type mice fed the same diet (Table 2). *Mdr2^−/−^* mice fed a 1% carbonyl iron diet also had significantly higher serum iron levels than *Mdr2^−/−^* mice fed a control diet. UIBC was higher in both wild-type and knockout animals fed an iron-deficient diet compared with those fed a high-iron diet. TIBC was increased in carbonyl iron-fed *Mdr2^−/−^* compared with wild-type mice fed the same diet. TS was lower in wild-type mice fed an iron-deficient diet compared with mice fed either a control or 1% carbonyl iron diet (Table 2), while *Mdr2^−/−^* mice fed an iron-deficient diet had lower TS than wild-type or knockout animals fed a carbonyl iron diet. Animals on an FVB background have inherently high iron, with previous reports indicating a transferrin saturation of 80% on a control diet [27].

**Table 2:** Hematological and serum iron-related parameters in wild-type and *Mdr2^−/−^* mice fed control, iron-deficient diet, or 1% carbonyl iron diets

Serum parameter	Genotype	Control diet	1% Carbonyl iron	Iron-deficient diet
**Hb (mg/dl)**	***Mdr2^+/+^***	12.9 ± 0.2	12.9 ± 0.2	8.4 ± 0.5
	***Mdr2^−/−^***	12.1 ± 0.2	13.2 ± 0.2	7.7 ± 0.5
Serum Iron (μg/dl)*	***Mdr2^+/+^***	310 ± 40	225 ± 49	135 ± 49
	***Mdr2^−/−^***	314 ± 30	641 ± 77^ab^	280 ± 103
UIBC (μg/dl)*	***Mdr2^+/+^***	69 ± 8	61 ± 10	185 ± 21^c^
	***Mdr2^−/−^***	199 ± 8^a^	51 ± 10^b^	247 ± 24
**TIBC (μg/dl)**	***Mdr2^+/+^***	379 ± 42	286 ± 50	296 ± 43
	***Mdr2^−/−^***	513 ± 33	692 ± 82	515 ± 85
**Transferrin**	***Mdr2^+/+^***	80 ± 2	73 ± 5	28 ± 6^c^
Saturation (%)*	***Mdr2^−/−^***	59 ± 2^a^	92 ± 1^ab^	34 ± 9^b^

Results are presented as mean ± SEM; *n* = 13–16. * Significant diet*Genotype interaction *P<*0.05. Post-hoc analysis: ^a^*P*<0.05 *Mdr2^+/+^* versus *Mdr2^−/−^* fed the same diet; ^b^*P*<0.05 compared with *Mdr2^−/−^* fed a control diet; ^c^*P*<0.05 compared with *Mdr2^+/+^* fed a control diet.

### Hepatic, splenic, and cardiac iron concentrations in wild-type and *Mdr2^−/−^* mice fed iron-deficient or iron-loaded diets

**Figure 5:**
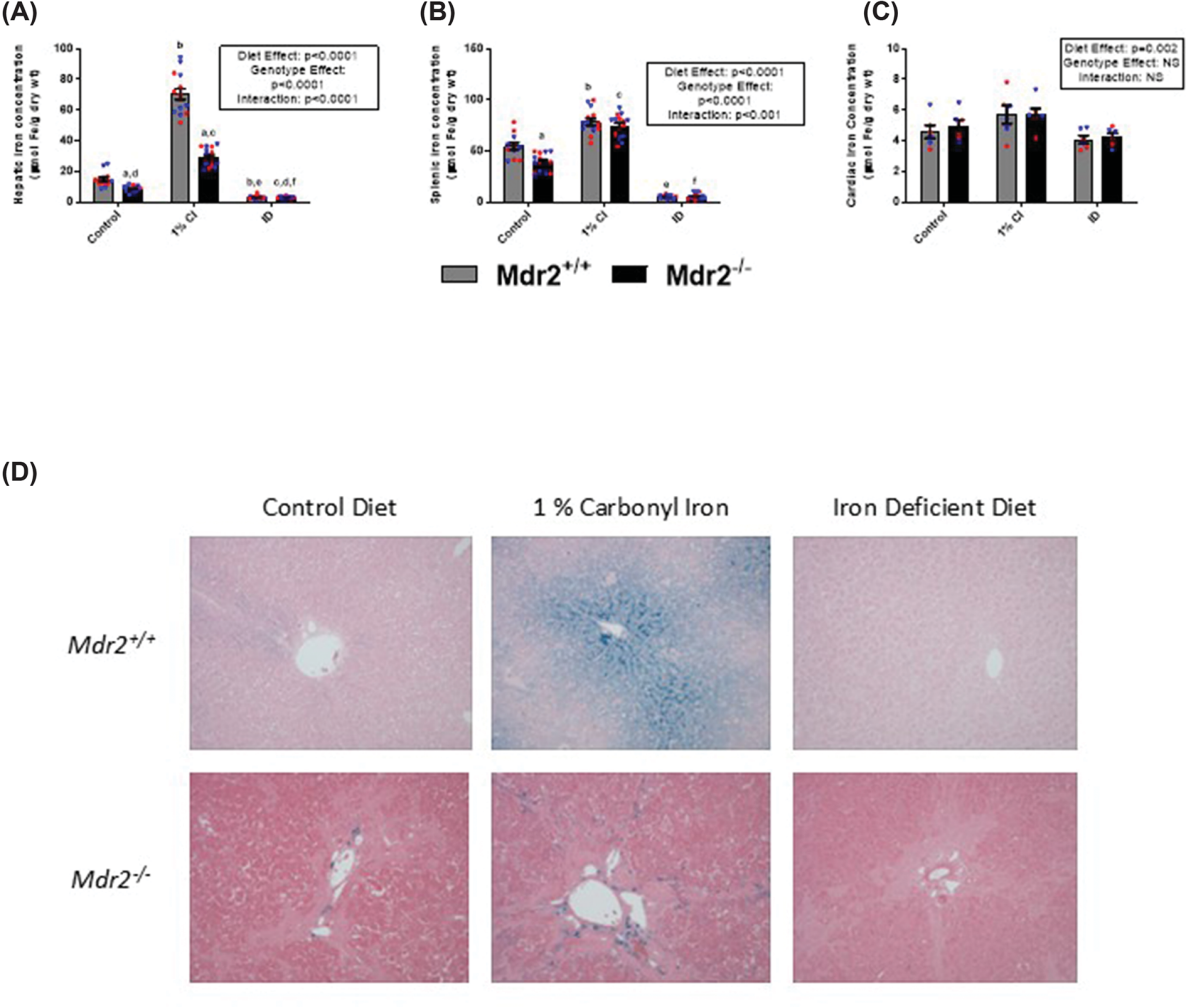
Hepatic, splenic, and cardiac iron stores in wild-type and Mdr2^−/−^ mice fed iron-loaded or iron-deficient diets Hepatic (**A**), splenic (**B**), and cardiac (**C**) iron concentrations were measured as described in the ‘Materials and methods’ section. Hepatic and splenic iron were significantly reduced in Mdr2^−/−^ animals compared with wild-type mice fed a control diet. Likewise, hepatic iron was reduced in knockout animals fed a carbonyl iron diet. Cardiac iron was similar in Mdr2^−/−^ versus Mdr2^+/+^ fed the same diet. Hepatic iron distribution (**D**) was assessed using Perls’ Prussian blue staining on paraffin-embedded sections. Mdr2^−/−^ mice were relatively resistant to hepatocyte iron loading when fed a 1% carbonyl iron. Original magnification: 100×. *n* = 13–16. Abbreviations: 1% CI—1% carbonyl iron, ID—iron-deficient diet. a*P*<0.05 versus wild-type on same diet; b*P*<0.05 versus wild-type on control diet; c*P*<0.05 versus Mdr2^−/−^ on control diet; d*P*<0.05 versus Mdr2^−/−^ on carbonyl iron diet; e*P*<0.05 versus wild-type on the control and carbonyl iron diets; f*P*<0.05 versus Mdr2^−/−^ on the control and carbonyl iron diets. Red symbols = male mice; blue symbols = female mice.

To determine whether *Mdr2^−/−^* mice fed an iron-loaded diet demonstrated hepatocyte iron loading, we measured tissue iron concentrations and assessed iron distribution (by Perls’ staining) in liver, spleen, and heart from mice challenged with high or low iron diets. When fed a control diet, *Mdr2^−/−^* mice had a significantly lower hepatic iron concentration (Figure 5A) than wild-type mice. Hepatic iron concentration increased significantly in both wild-type and *Mdr2^−/−^* mice fed 1% carbonyl iron relative to a control diet and was significantly higher in wild-type mice than in *Mdr2^−/−^* mice. Feeding an iron-deficient diet resulted in decreases in hepatic iron in both wild-type and *Mdr2^−/−^* mice, and levels did not differ significantly between wild-type and *Mdr2^−/−^* mice (3.1 ± 0.3 versus 3.0 ± 0.2 μmol Fe/g dry wt.). Splenic iron concentration (Figure 5B) was also significantly lower in *Mdr2^−/−^* mice fed a control diet when compared with wild-type mice (38.8 ± 2.3 versus 51.5 ± 4.5 μmol Fe/g dry wt., *P* < 0.05). When fed 1% carbonyl iron, there was a significant increase in splenic iron concentration in both wild-type and *Mdr2^−/−^* mice compared with control diet and splenic iron concentration was significantly lower in wild-type and *Mdr2^−/−^* mice following an iron-deficient diet (Figure 5B) when compared with both control diet and carbonyl iron diet groups. Cardiac iron was similar in Mdr2^+/+^ and Mdr2^−/−^ animals (Figure 5C). Similar to the results described above for the time course study, stainable hepatocellular iron was not seen in *Mdr2^−/−^* mice, and the iron staining that was observed was in the RE system (Figure 5D). While both wild-type and *Mdr2^−/−^* mice had more stainable hepatic iron on an iron-loaded diet, the cellular pattern of iron loading differed between genotypes. Wild-type mice showed significant hepatocellular iron accumulation, while the iron that was present in the knockout animals was predominantly in Kupffer cells (Figure 5D). This may indicate that *Mdr2^−/−^* were resistant to hepatocyte iron loading when fed 1% carbonyl iron. Both wild-type and *Mdr2^−/−^* mice fed an iron-deficient diet had no stainable hepatic iron.

### *Mdr2^−/−^* mice fed diets of differing iron content have altered hepatic expression of iron-regulatory genes and proteins

**Figure 6:**
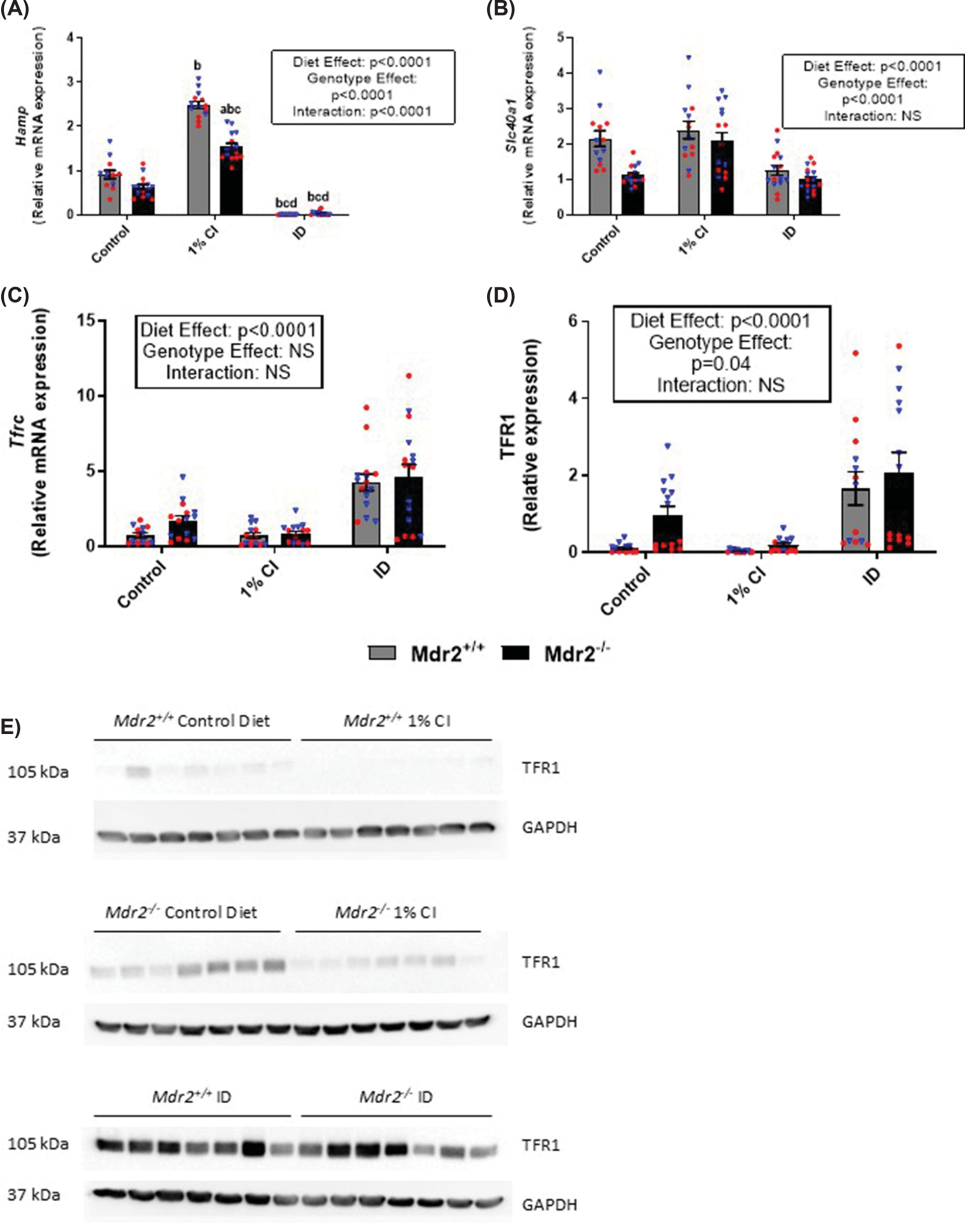
Hepatic expression of iron-regulatory genes and proteins Hepatic Hamp (**A**), Slc40a1 (**B**), and Tfrc (**C**) mRNA levels were measured using RT-PCR. TFR1 results were confirmed at the protein level using Western blotting (**D,E**). Hamp and Slc40a1 expression was lower in Mdr2^−/−^ mice compared with wild-type mice fed CI and control diets, respectively. There were no significant changes in Tfrc expression between Mdr2^−/−^ and wild-type mice on the same diet. Results are presented as mean ± SEM; *n* = 13–16. a*P* < 0.05 versus wild-type on same diet; bP <0.05 versus wild-type on control diet; c*P* < 0.05 versus Mdr2^−/−^ on control diet; d*P* <0.05 versus wild-type and Mdr2^−/−^ on carbonyl iron diet. Red symbols = male mice; blue symbols = female mice.

Given the differences observed in hepatic iron accumulation in wild-type and knockout mice, we next sought to investigate if hepatic expression of iron-related genes was dysregulated in mice with cholestasis when challenged by different iron diets. Hepatic *Hamp* mRNA expression (Figure 6A) was significantly lower in *Mdr2^−/−^* mice fed either control or 1% carbonyl iron diets compared with wild-type mice fed the same diet (1.5-fold and 1.6-fold, respectively). In both wild-type and *Mdr2^−/−^* mice, *Hamp* expression increased significantly following feeding with 1% carbonyl iron (2.7-fold and 2.5-fold). In contrast, *Hamp* mRNA was almost undetectable in wild-type mice fed an iron-deficient diet and was reduced 20.7-fold in *Mdr2^−/−^* mice fed an iron-deficient diet relative to the same strain on a control diet. Hepatic *Slc40a1* mRNA expression (Figure 6B) was reduced in knockout animals on a control diet compared with wild-type animals on the same diet. Maintaining mice on a carbonyl iron diet has no effect on *Slc401* expression in wild-type mice but increased expression in *Mdr2^−/−^* mice to levels comparable to that of the wild-type animals. *Slc40a1* decreased with an iron-deficient diet compared with a control diet. Both *Tfrc* mRNA (Figure 6C) and protein (Figure 6D) expression increased on an iron-deficient diet. TFR1 protein expression increased in *Mdr2^−/−^* mice fed a control diet compared with wild-type animals on control diet, but genotype had no effect on *Tfrc* expression. To determine whether cellular uptake of NTBI was altered following an iron challenge, we examined expression of *Slc39a14* (ZIP14); however, no significant effect of diet or genotype was observed (Supplementary Figure S5).

**Figure 7:**
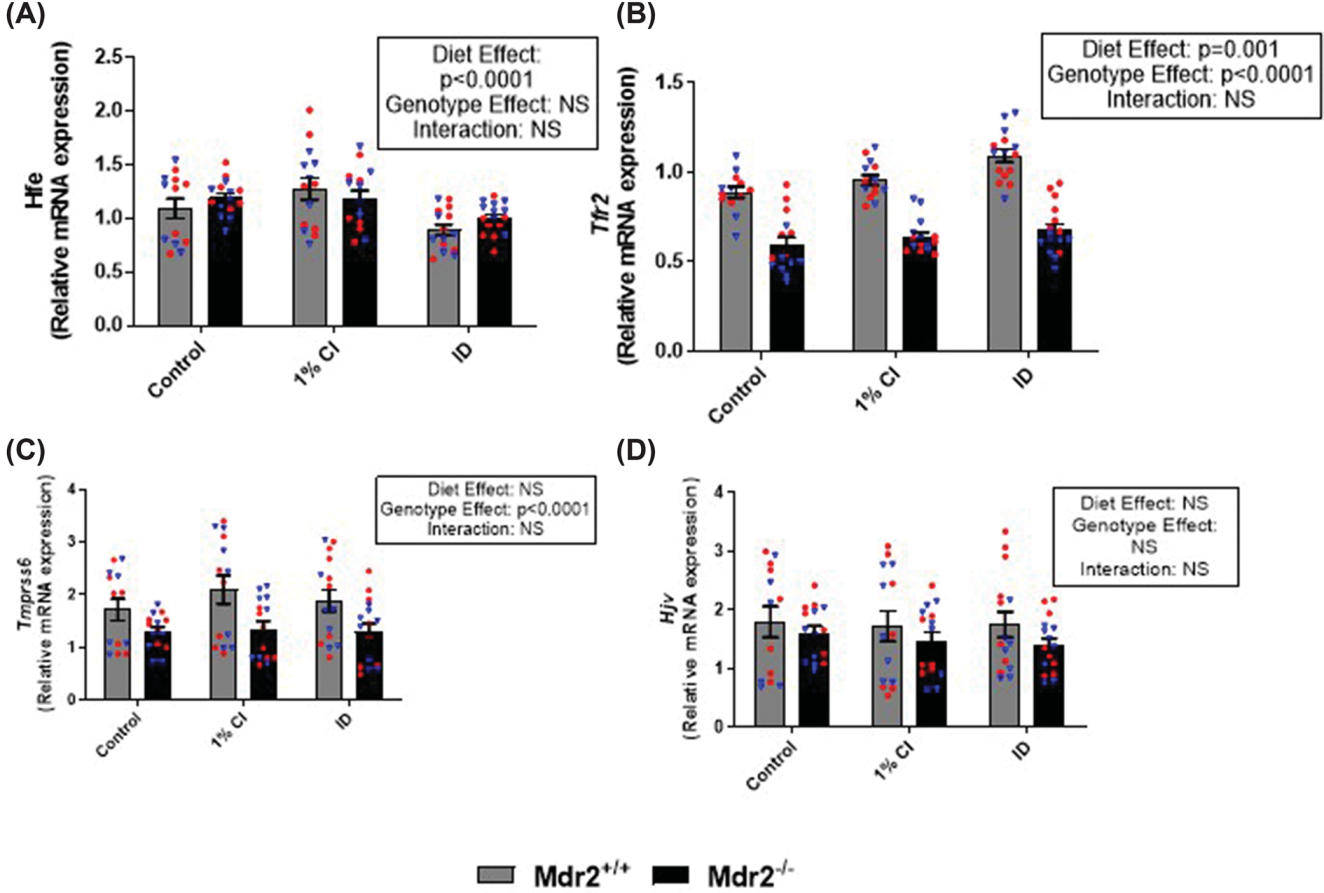
Expression of genes encoding proteins that regulate hepcidin transcription Hfe (**A**) mRNA levels were not significantly different in Mdr2^−/−^ mice compared with wild-type mice, while Tfr2 (**B**) mRNA levels were decreased in Mdr2^−/−^ mice on all diets. Tmprss6 mRNA expression (**C**) was lower in Mdr2^−/−^ mice than in wild-type mice, while Hjv expression (**D**) did not differ with diet or genotype. Results are presented as mean ± SEM from *n* = 13–16. Red symbols = male mice; blue symbols = female mice.

*Hfe* mRNA expression (Figure 7A) was similar in wild-type and *Mdr2^−/−^* mice regardless of diet but decreased in both strains with an iron-deficient diet. In contrast, *Mdr2^−/−^* mice had lower *Tfr2* mRNA expression than wild-type mice on each diet (Figure 7B). *Mdr2^−/−^* mice had lower *Tmprss6* expression (Figure 7C) than wild-type mice. There were no significant changes in *Hjv* mRNA levels with either diet or genotype (Figure 7D). These results suggest that cholestasis affects the iron regulatory machinery in the liver of *Mdr2^−/−^* mice.

## Discussion

One potential mechanism for explaining the enhanced iron accumulation that is often found in advanced liver disease is that hepatic synthetic dysfunction results in reduced hepcidin levels, leading to inappropriately high duodenal iron absorption and excess iron deposition within the liver [19]. However, unlike hepatocellular liver disease, hepatic iron accumulation is comparatively rare among patients with cholestatic liver injury [8,9]. To better understand this dichotomy, we investigated iron homeostasis in the *Mdr2^−/−^* mouse model of cholestasis and hepatic fibrosis. *Mdr2^−/−^* mice had lower hepatic iron stores than their wild-type counterparts, with hepatic iron predominantly located in macrophages and Kupffer cells in the *Mdr2^−/−^* mice. Iron depletion appeared to be a specific feature of the liver, as splenic and cardiac iron concentrations were similar in wild-type and *Mdr2^−/−^* mice.

Our studies have shown disturbances in iron homeostasis in *Mdr2^−/−^* mice at all ages studied, regardless of the level of hepatic dysfunction. Thus, this aberrant iron homeostasis (specifically hepatic iron deficiency) is unlikely to be due to liver injury itself and is either the direct result of *Mdr2* absence (via mechanisms that are currently unknown) or reflects the associated cholestasis. Iron deficiency and perturbations in bile acids have also been described in gastric carcinogenesis [28], and altered bile acids in cholestasis can impact hepcidin synthesis [29]. In addition, the localisation of hepatic iron in *Mdr2^−/−^* mice to the RE cells likely reflects enhanced hepatic necroinflammatory activity [10]. Iron deposition in non-parenchymal cells including macrophages, Kupffer cells, and endothelial cells has been associated with more advanced injury in both NAFLD and HCV [10,12]. Thus, further investigation of the importance of iron partitioning in cholestatic injury and the role it may play in the progression of hepatic fibrosis and disease evolution is warranted.

The present study has confirmed many of the findings of Kolouchova et al. [20], who demonstrated that bile duct-ligated rats have reduced hepatic iron stores, lower hepcidin, and increased serum iron levels. This group attributed the hepatic iron deficiency to inappropriate hepatic iron export due to increased expression of FPN1. In our study, however, hepatic FPN1 expression was lower in *Mdr2^−/−^* mice than in wild-type mice, and thus the hepatic iron deficiency was unlikely to be due to increased iron export via FPN1. An alternative mechanism may be that Mdr2 deficiency or cholestasis impairs hepatocellular iron uptake. Huang et al. [29] have suggested that hydrophobic bile acids in cholestasis suppress interleukin 6-induced STAT3 phosphorylation, and this results in reduced hepcidin levels.

When *Mdr2^−/−^* mice were fed a high-iron diet, both their serum iron levels and TS increased compared with *Mdr2^−/−^* mice fed a control diet. Wild-type mice accumulated large amounts of hepatic iron on the iron-loaded diet, and Perls’ Prussian blue staining indicated that this iron accumulation was predominantly in Zone I and II hepatocytes. Although *Mdr2^−/−^* mice fed a high-iron diet had increased hepatic iron stores, there was significantly less iron accumulation than in wild-type mice, and iron deposition occurred primarily in Kupffer cells, rather than hepatocytes. The high levels of serum iron in *Mdr2^−/−^* mice, combined with a relative apparent resistance to hepatocellular iron accumulation, suggest that the hepatic iron deficiency in *Mdr2^−/−^* mice may be due to impaired hepatocellular iron uptake.

Hepatocellular iron uptake occurs, in part, via receptor-mediated endocytosis in clathrin-coated vesicles following an interaction between holotransferrin in the circulation and transferrin receptor 1 on the hepatocyte sinusoidal surface [30]. Once iron is delivered from endocytic vesicles to the cytoplasm, the receptors are returned to the cell surface in recycling vesicles [31]. Cholestatic conditions cause a number of changes in the hepatocyte cytoskeleton, such as disruption of microtubules, increased intermediate filaments, and accumulation of disorganised bundles of actin microfilaments in the pericanalicular region [32]. In addition, in experimental systems, bile salts inhibit the action of molecular motors (kinesin and dynein), which move vesicles along microtubules [33]. This has mostly been studied in the context of vesicular trafficking of canalicular proteins to the appropriate region of the plasma membrane and contributes to the altered hepatocyte polarity seen in cholestasis [34–36]. Given that the transferrin cycle relies on vesicular movement along the microtubule system, it is conceivable that alterations in intracellular trafficking associated with cholestasis could influence aspects of transferrin receptor-mediated iron handling. However, in our study, *TfR1* expression appeared to respond appropriately to cellular iron stores, providing no direct evidence of impaired transferrin receptor-mediated iron uptake. Therefore, the extent to which cholestasis affects transferrin receptor-mediated iron uptake remains unclear and warrants further investigation. We also examined if reduced hepatocellular iron in our model may also be the result of impaired hepatocellular uptake of NTBI, which is mediated by ZRT/IRT-like Protein 14 (ZIP14 or SLC39A14) [37]. In our study, *Slc39a14* expression was not altered following an iron challenge; however, down-regulation of ZIP14 has been associated with other chronic liver diseases [38].

Both wild-type and *Mdr2^−/−^* mice were able to regulate *Hamp* expression in response to iron challenges. However, many mediators of *Hamp* expression, such as TFR2 and TMPRSS6, were differentially expressed between wild-type and *Mdr2^−/−^* mice. Tmprss6 can be regulated by iron status, with increased *Tmprss6* expression observed following chronic iron treatment and in states of iron deprivation [39,40]. These authors suggest that in addition to TMPRSS6 being stimulated by iron deficiency, in iron overload TMPRSS6 acts as a negative feedback inhibitor to prevent excessive hepcidin release [39]. In our study *Tmprss6* expression was increased in wild-type mice following a carbonyl iron diet and an iron-deficient diet. The decreased *Tmprss6* expression observed in knockout animals may reflect the increased inflammation observed in *Mdr2^−/−^* mice, given that inflammatory conditions have been shown to result in reduced *Tmprss6* levels [41,42].

The present study is the first to characterise altered iron homeostasis in the *Mdr2^−/−^* mouse model of cholestasis. Overall, the data suggest that cholestatic conditions are associated with a resistance to hepatocyte iron loading and explain, in part, the lower hepatic iron stores in patients with cholestatic diseases compared with hepatocellular diseases. In the context of this hepatic iron uptake resistance, the serum iron indices in cholestasis should be interpreted with some caution since they indicate normal or increased mobilizable iron when in fact mobilizable hepatic iron stores are reduced. A full understanding of the mechanisms underlying the effects on iron metabolism requires further study.

## Materials and methods

### Animals

FVB.129P2-*Abcb4^tm1Bor^*/J (*Mdr2^−/−^*) mice were obtained from The Jackson Laboratory (Bar Harbour, Maine). Wild-type control mice (FVB/n) were purchased from the Animal Resource Centre (Perth, Western Australia). Animals were housed under a 12-h light/dark cycle with *ad libitum* access to water and a standard rodent diet (Rat and Mouse Cubes; Specialty Feeds, Glen Forrest, Western Australia). Animals were housed at the QIMR Berghofer Animal Facility, Herston, and the University of Queensland Biological Resources Facility, Woolloongabba, where experimental procedures were performed. No anaesthetics were used; animals were killed using CO_2_ asphyxiation at the completion of the study. To kill animals, 100% CO_2_ is administered at 2.3 l/min to achieve 30% chamber volume displacement/minute. Wild-type and *Mdr2^−/−^* mice were studied at 3, 5, 8, 12, and 16 weeks of age. Equal numbers of male and female mice were included in each group.

### Iron challenges

Wild-type and *Mdr2^−/−^* mice were fed either a control, an iron-deficient, or a 1% carbonyl iron diet. Diets were prepared as previously described [43–45]. Mice were fed either a control diet from 3 to 9 weeks of age, an iron-deficient diet from 3 to 9 weeks of age, or a control diet from 3 to 5 weeks of age followed by 1% carbonyl iron from 5 to 9 weeks of age. The two cohorts (time course (described above) and iron challenge) were fed different control diets, as the iron challenge diets were not commercially sourced. The iron concentration was 70 mg/kg versus 107 mg/kg (time course versus iron challenge, respectively).

### Blood and serum analysis

Serum iron, unsaturated iron-binding capacity, TIBC, and TS were measured using an Iron/TIBC kit (Pointe Scientific, Canton, MI, USA). Hb was measured using a QuantiChrom™ Haemoglobin Assay Kit (BioAssay Systems). Samples were assayed according to the manufacturer’s instructions. Serum ferritin was measured using a Mouse Ferritin ELISA Kit (Kamiya Biomedical, Seattle, WA, USA) according to the manufacturer’s instructions. Serum samples were assayed in duplicate. Full blood counts were performed on whole blood using a Coulter® Ac.T diff Counter (Beckman Coulter, USA). Serum liver function tests (alanine transaminase, aspartate transaminase, alkaline phosphatase, and bilirubin) were measured using a colourimetric assay (BIOO Scientific, Austin, TX) as per the manufacturer’s instructions.

### Tissue iron analysis

Hepatic, splenic, and cardiac iron concentrations of dried tissue were measured colourimetrically as described by Torrance and Bothwell [46].

### Liver and spleen histology

Haematoxylin and eosin, Picrosirius red, and Perls’ Prussian blue staining were performed on paraffin-embedded liver and spleen sections as previously described [47,48].

### Real-time RT PCR

qRT-PCR was used to analyse gene expression as described previously [49]. Genes studied included *Hamp1* (the gene encoding hepcidin), *Tfrc* (the gene encoding transferrin receptor 1), *Tfr2* (the gene encoding transferrin receptor 2), *Slc40a1* (the gene encoding ferroportin 1), *Cybrd1* (the gene encoding duodenal cytochrome B), *Hfe* (the gene encoding human homeostatic iron regulator protein), *Tmprss6* (the gene encoding matriptase 2), *Hjv* (the gene encoding hemojuvelin), *Coll1a1* (procollagen type 1), *Pdgfrb* (platelet-derived growth factor receptor B), *Timp1* (tissue inhibitor of metalloproteinase 1), and *Slc39a14* (ZRT/IRT-like Protein 14). Gene expression was normalised to *B2m* (the gene encoding beta-2-microglobulin) mRNA levels. Primer sequences are found in Supplementary Table S1.

### Western blotting

Liver tissue (50–100 mg) was homogenised in 1 ml cold (4°C), freshly prepared HES+ extraction buffer (20 mM Hepes, 1 mM EDTA, 250 mM sucrose, 2 mM Na_3_VO_4_, 10 mM NaF, 1 mM Na_4_P_2_O_7_, 0.5 mM PMSF, pH 7.6) containing protease inhibitor cocktail (Roche) using a TissueRuptor (Qiagen) with disposable probes. Protein levels were quantified using a BCA Protein Assay Reagent Kit (Pierce, Rockford, IL, USA) according to the manufacturer’s instructions. Total liver protein homogenates (30 μg) were electrophoresed on a 10% SDS PAGE and transferred to a PVDF membrane. Membranes were blocked for 1 h at room temperature in blocking buffer (10% skim milk powder in TBS with 0.1% Tween-20). Primary antibodies (L-ferritin (1:2000) (Sigma), TFR1 (1:2000) (Sigma), TFR2 [50], FPN1, and GAPDH (1:150,000) (Millipore)) were applied at optimal concentration in blocking buffer overnight at 4°C. The membranes were then incubated with the secondary antibodies (anti-mouse or rabbit HRP, 1:200,000 and 1:100,000, respectively) after washing with TBST (Tris-buffered saline 0.1% Tween-20). ECL substrate (Pierce) was applied for 5 min, and chemiluminescence was detected and photographed using an Image Station 4000MM Pro (Carestream). Gels were analysed by densitometry, and the expression of target proteins was normalised to GAPDH levels.

### Hydroxyproline assay

A hydroxyproline assay was used to measure hepatic collagen content in the present study and was performed according to the methods of Jonsson et al. [51].

### Statistical analysis

Statistical analysis was performed using IBM SPSS Statistics 27. Timecourse data was analysed using a two-way ANOVA to test the influence of age and genotype individually and to assess the age*genotype interaction. Data from the iron challenge experiments was analysed using a two-way ANOVA to test the overall effects of genotype and diet and to observe any diet*genotype interaction. Where a significant interaction was noted during two-way ANOVA analysis, LSD post-hoc analysis was performed to assess differences between individual groups. Where the interaction was not significant, the role of the main effects was reported; however, if the interaction was significant, results from the post-hoc analysis were reported. For all analyses, *P*<0.05 was considered statistically significant.

## Supplementary Material

Supplementary Figures S1-S5 and Table S1

## Data Availability

All data generated or analysed during this study are included in this article. Further inquiries can be directed to the corresponding authors.

## Open Access

Open access for this article was enabled by the participation of the University of Queensland in an all-inclusive Read & Publish agreement with Portland Press and the Biochemical Society under an agreement with CAUL.

## Abbreviations

ALD: alcoholic liver disease
ANOVA: analysis of variance
BDL: bile duct ligation
CI: carbonyl iron
Cybrd1: duodenal cytochrome b
ELISA: enzyme-linked immunosorbent assay
FPN1: ferroportin 1
GAPDH: glyceraldehyde-3-phosphate dehydrogenase
Hamp/Hamp1: gene encoding hepcidin
Hb: haemoglobin
HCV: hepatitis C virus
HFE: human homeostatic iron regulator protein
HJV: hemojuvelin
LSD: least significant differences
*Mdr2* ^−/−^: multidrug-resistance 2
NAFLD: nonalcoholic fatty liver disease
NASH: non-alcoholic steatohepatitis
NTBI: non-transferrin-bound iron
PVDF: polyvinylidene fluoride
RT-PCR: reverse transcription polymerase chain reaction
SDS-PAGE: sodium dodecyl sulfate polyacrylamide gel electrophoresis
Slc40a1: gene encoding ferroportin 1
TF: transferrin
TFR: transferrin receptor
Tfrc: gene encoding transferrin receptor 1
TIBC: total iron-binding vapacity
TMPRSS6: transmembrane protease serine 6 (matriptase-2)
TS: transferrin saturation
RE: reticuloendothelial
UIBC: unsaturated iron-binding capacity
ZIP14: ZRT/IRT-like protein 14
