# Supplementary material for: Hepatic iron accumulation is reduced in the cholestatic Mdr2−/− mouse
Source: Biosci Rep. 2026 Jul 22;46(8):BSR20250360. doi: 10.1042/BSR20250360 (PMC13402865; doi:10.1042/BSR20250360)

Supplementary Table 1: Primer sequences used for qRT-PCR:

**Gene**	**Forward Primer (5'-3')**	**Reverse Primer (5'-3')**
Hamp	TTGCGATACCAATGCAGAAG	GGATGTGGCTCTAGGCTATGTT
Tfrc	GAGGCAGACCTTGCACTCTT	TGACTGAGATGGCGGAAAC
Tfr2	GAGCCACAGAGTGGTTGGAG	GTCCAGGCTCACGTACACAA
Slc40a1	GCCACTGCGATCACAATCC	CTGGTCAATCCTTCTAATGGTAGCAT
Cybrd1	GCAGCGGGCTCGAGTTA	TTCCAGGTCCATGGCAGTCT
Tmprss6	CATCAACTTCACCTCCCAGA	CAGTCCATTCACAGAGCAGAG
Hfe	AGGTCACGAAGTTGGGAGTG	AGCCGCTGGTACTGTTGTCT
Hjv	TTGACCTCGGGAAACATCAC	GACTGGCCCATACCTATCCA
Coll1a1	TCCGGCTCCTGCTCCTCTTA	GTATGCAGCTGACTTCAGGGATGT
Pdgfrb	TCCCACATTCCTTGCCCTT	TCGCTACTTCTGGCTGTCGAT
Timp1	TCCTCTTGTTGCTATCACTGATAGCTT	CGCTGGTATAAGGTGGTCTCGTT
Slc39a14	CTGGCTATTGGTGCCTCCTTCA	TGCCAGCATTGAGCAGGATGAC

Supplementary Figure 1: Liver histology in wild-type and *Mdr2*$^{-/-}$ mice from 3 to 16 weeks of age.

Representative liver sections stained with Sirius red (A-F) and haematoxylin and eosin (G-L) from wild-type (A and G) and *Mdr2*$^{-/-}$ mice at 3 (B and H), 5 (C and I), 8 (D and J), 12 (E and K) and 16 (F and L) weeks of age. Original magnification: 100X. n = 7-8.

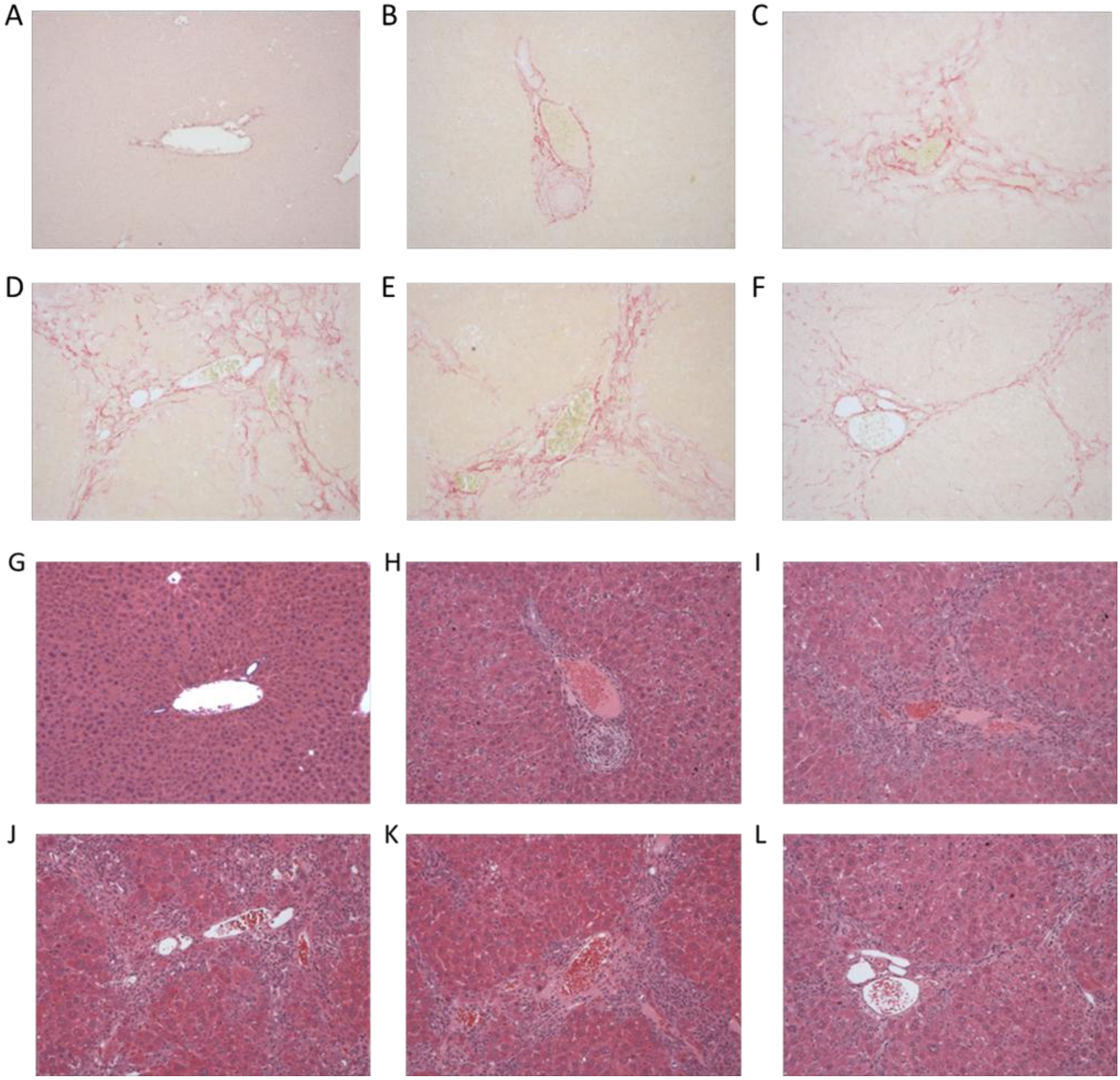

Supplementary Figure 2: Liver function in wild-type and *Mdr2$^{-/-}$* mice.

Alanine aminotransferase (A), aspartate aminotransferase (B) and alkaline phosphatase (C) and bilirubin (D) were upregulated in knockout animals compared with controls. Results are presented as mean ± SEM from n = 4-8. Red symbols = male mice; blue symbols = female mice.

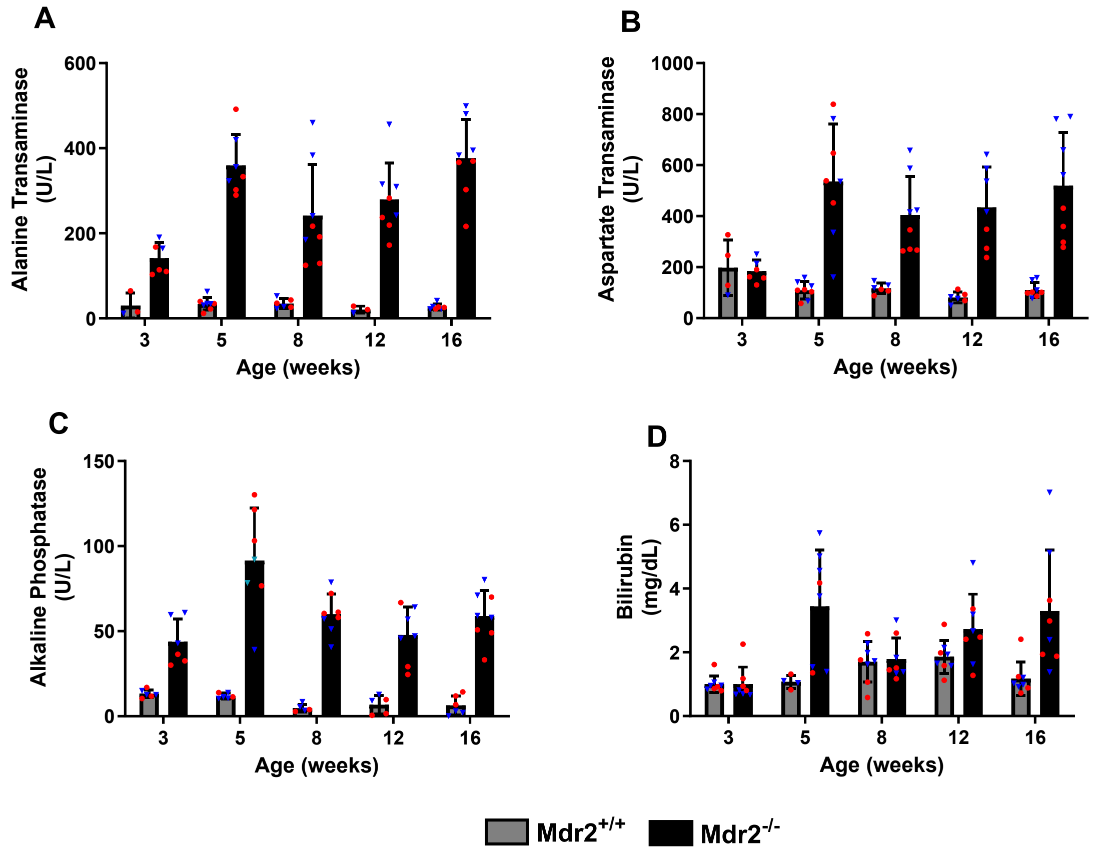

Supplementary Figure 3: Hepatic hydroxyproline and expression of fibrogenic genes in wild-type and *Mdr2*$^{-/-}$ mice.

Hepatic hydroxyproline (A) and expression of fibrogenic genes (procollagen type 1 (B), platelet derived growth factor receptor B (C) and tissue inhibitor of metalloproteinase 1 (D)) are increased in knockout animals at 3, 5, 8, 12 and 16 weeks. Results are presented as mean ± SEM from n = 8. Red symbols = male mice; blue symbols = female mice.

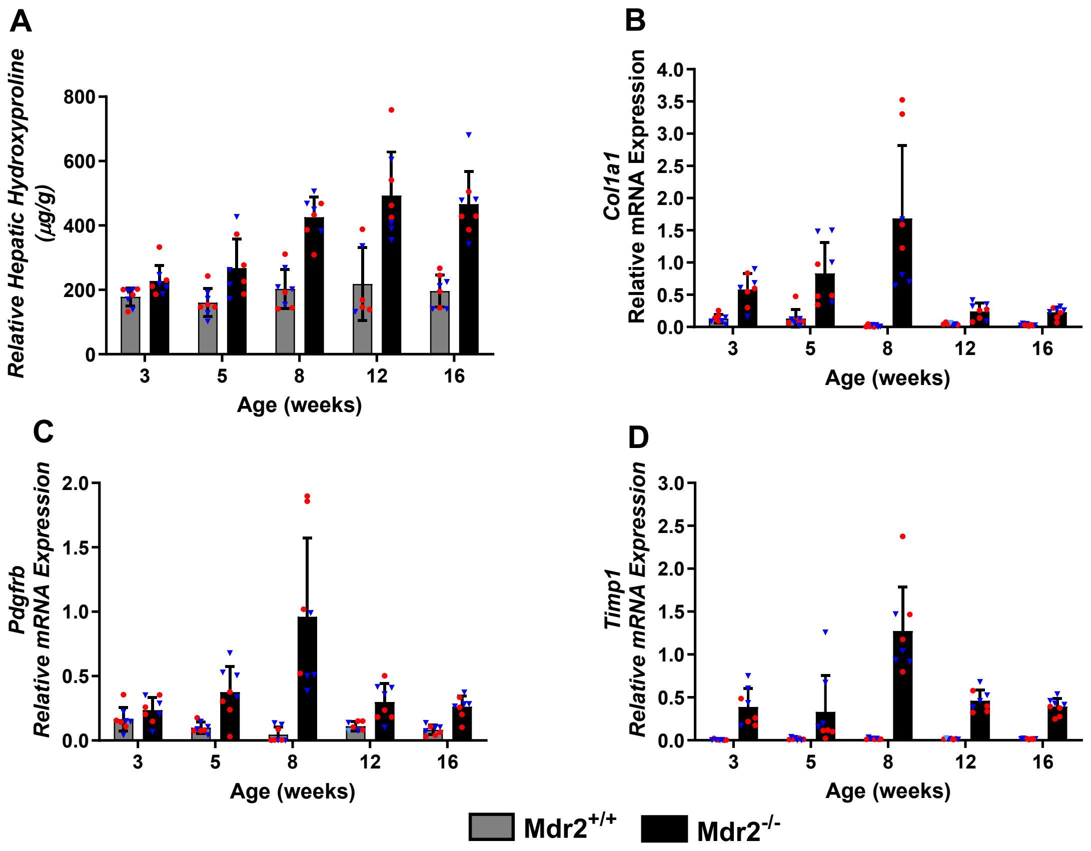

Supplementary Figure 4: Splenic and cardiac iron stores in wild-type and *Mdr2*$^{-/-}$ mice.

A and B: Splenic and cardiac iron concentrations increased with age in wild-type and *Mdr2*$^{-/-}$ mice but did not differ significantly with genotype. Results are presented as mean ± SEM from n = 8. C: Splenic iron distribution was assessed by Perls' Prussian blue staining. Original magnification: 100X. WP = white pulp; RP = red pulp. Red symbols = male mice; blue symbols = female mice.

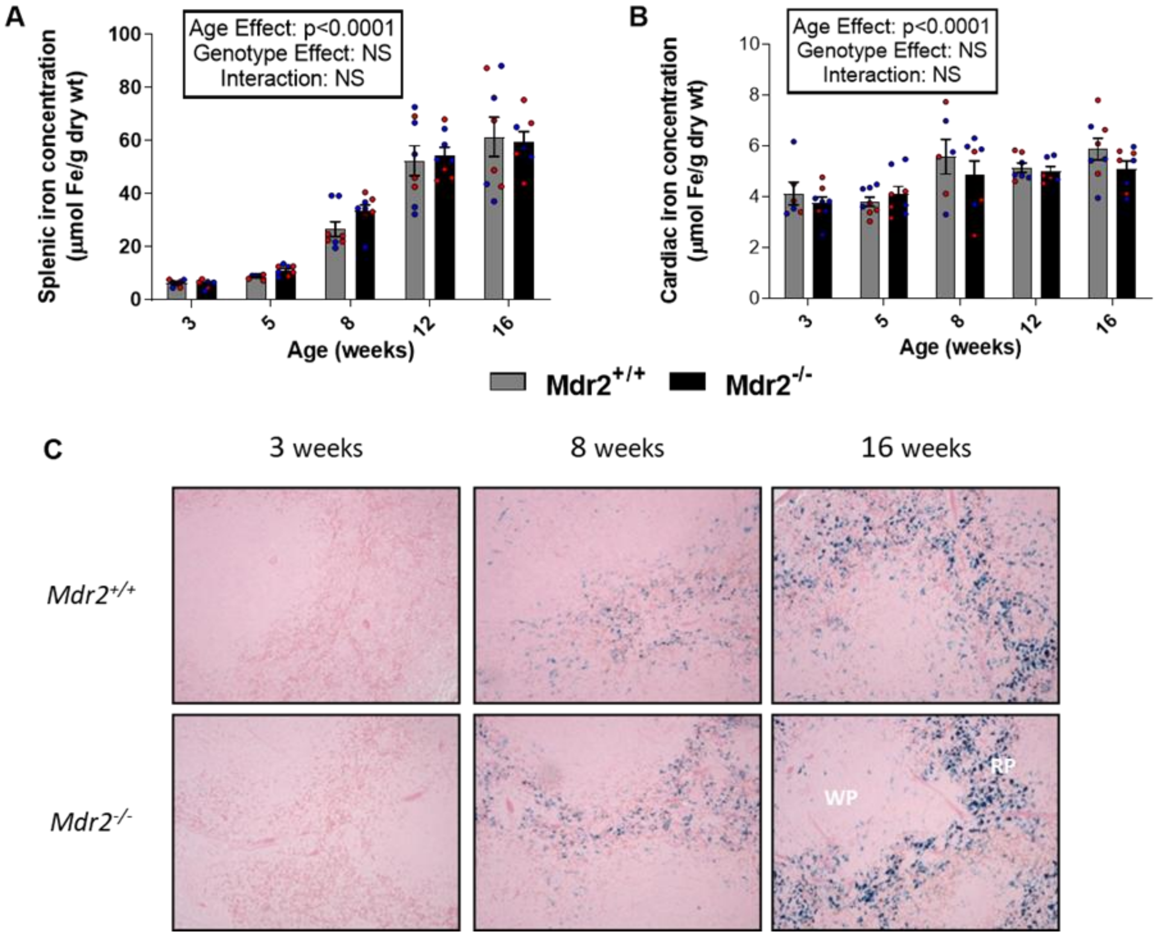

Supplementary Figure 5: Hepatic expression of ZRT/IRT-like Protein 14 (Zip14 or *Scl39a14*) following iron challenge in wild-type and *Mdr2*$^{-/-}$ mice.

Hepatic expression of *Scl39a14* was not significantly changed following iron challenge in wild-type of *Mdr2*$^{-/-}$ mice. Results are presented as mean ± SEM from n = 6-12. Red symbols = male mice; blue symbols = female mice.

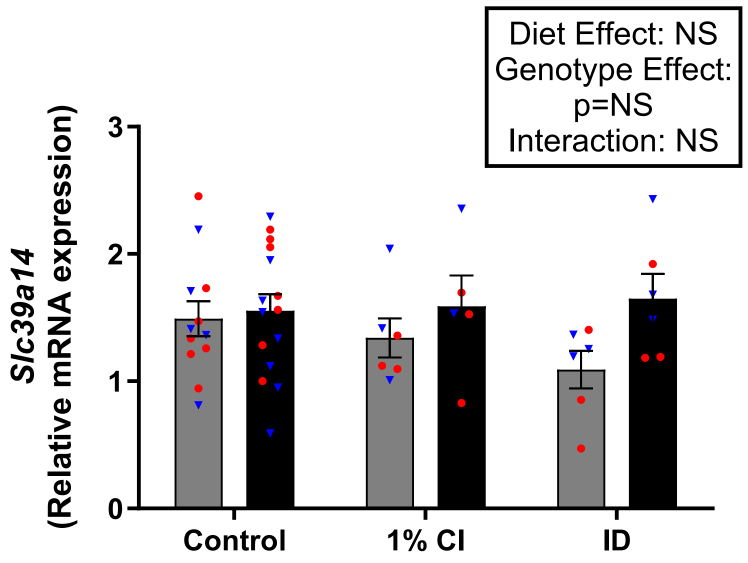

Supplement: Supplementary Figures S1-S5 and Table S1 [file BSR-2025-0360_supp.pdf]
